# Biopsy-Proven Glomerular Diseases in Saudi Arabia: The Clinicopathological Spectrum and Predictors of Renal Outcomes in a Single-Center Cohort and Four Decades of National Data

**DOI:** 10.7759/cureus.118097

**Published:** 2026-10-18

**Authors:** Hassan M Homadi, Muhammad Abdul Mabood Khalil, Nihal M Sadagah, Hinda Hassan Mahmood, Abeer A AlMarhabi, Alfatih A Altom, Turki A Hamdi, Ammar T Elgadi, Nadin A Alharbi, Bodoor Alahmadi, Wafaa M Alalyani, Salem Al-Qurashi

**Affiliations:** 1 Renal Diseases and Transplantation Centre, King Fahad Armed Forces Hospital, Jeddah, SAU; 2 Nephrology, Al-Noor Specialist Hospital, Makkah, SAU; 3 Department of Medicine, King Fahad Armed Forces Hospital, Jeddah, SAU; 4 Faculty of Medicine, University of Khartoum, Khartoum, SDN

**Keywords:** chronic kidney disease (ckd), diabetic kidney disease, egfr decline, focal segmental glomerulosclerosis, glomerulonephritis, interstitial fibrosis, kidney biopsy, lupus nephritis, renal outcomes, saudi arabia

## Abstract

Background

Glomerular diseases are a significant contributor to chronic kidney disease (CKD) in Saudi Arabia. Local data linking biopsy findings to renal outcomes are scarce, and the available series are mostly descriptive.

Objectives

The objective of this study is to characterize the clinicopathological spectrum of biopsy-proven glomerular disease at a single Saudi tertiary center, identify histological and clinical predictors of renal function decline at one year, and secondarily compare these findings descriptively with previously published Saudi biopsy series.

Methods

We retrospectively reviewed native kidney biopsies performed over a 24-year period. The primary outcome was a composite renal endpoint at one year (≥ 40% decline in eGFR or eGFR < 15 mL/min/1.73 m²), with sensitivity analyses using a 30% decline threshold. Predictors were assessed by multivariable logistic regression in a complete-case cohort of 289 patients.

Results

Of 700 biopsies screened, 333 met the inclusion criteria. The most common diagnoses were lupus nephritis (72; 21.6%), followed by focal segmental glomerulosclerosis (FSGS) (60; 18%), diabetic nephropathy (46; 13.8%), and IgA nephropathy (35; 10.5%). FSGS was the most common primary glomerular lesion, and lupus nephritis was the leading secondary diagnosis. Overall, 124 of 314 patients with follow-up (39%) reached the composite outcome at one year. Moderate to severe interstitial fibrosis and tubular atrophy (IFTA) (aOR 2.13, 95% CI 1.14-3.98; p = 0.018) and moderate to severe arteriosclerosis (aOR 2.38, 95% CI 1.09-5.26; p = 0.034) independently predicted the outcome, as did higher 24-hour urinary protein (aOR 1.08 per g/day, 95% CI 1.01-1.15; p = 0.026) and lower baseline eGFR (aOR 0.98 per mL/min/1.73 m², 95% CI 0.97-0.99; p < 0.001).

Conclusion

In this 24-year biopsy cohort, chronic histopathological damage was an independent predictor of renal function decline at one year, alongside proteinuria and baseline eGFR. These findings show that chronicity on biopsy carries prognostic weight and support the need for dedicated national glomerulonephritis (GN) registries combining histopathological findings with longitudinal follow-up.

## Introduction

Chronic kidney disease (CKD) is a growing public health burden in Saudi Arabia. Recent population data estimate a CKD prevalence of 4.76%, with most affected individuals at stage 3 [1]. In the Global Burden of Disease 2021 analysis, Saudi Arabia had the highest age-standardized CKD incidence rate of any country worldwide (495.8 per 100,000) [2]. Progression to end-stage kidney disease has risen steadily: in 2020, more than 20,000 patients were on dialysis, alongside approximately 7,273 living kidney transplant recipients [3]. This trajectory is driven by a high prevalence of hypertension and diabetes. Adult diabetes prevalence in Saudi Arabia reached 23% among those aged 20-79 years in 2024, one of the highest rates worldwide [4], with national meta-analyses estimating figures approaching 28% [5].

Within this landscape, glomerulonephritis (GN) is an important and potentially treatable cause of CKD and end-stage kidney disease, and percutaneous kidney biopsy remains the cornerstone of diagnosis, defining clinicopathological subtypes, guiding therapy, and refining prognosis. However, the distribution of biopsy-proven GN is not uniform and varies with ethnicity, geography, referral patterns, biopsy indication, and time, which limits how findings from one population can be applied to another [6]. Understanding the local spectrum is therefore essential to rational nephrology practice.

Over the past four decades, several Saudi biopsy studies have described the pattern of native kidney disease [7-25], from early series in the 1980s [7] through to more recent multicenter analyses [22,23]. Collectively, they point to focal segmental glomerulosclerosis (FSGS) as the commonest primary glomerular disease and lupus nephritis as the commonest secondary one, with regional variation and a rising contribution from diabetic kidney disease over time. These reports differ in method, however, with most being single-center retrospective series using varied classification systems and study populations. Most are also cross-sectional and do not report renal outcomes. An analysis comparing current single-center outcomes with the national literature is lacking.

A related challenge arises in patients with diabetes, in whom kidney disease is often presumed to be diabetic in origin without histological confirmation [26]. Biopsy studies have shown this assumption to be unreliable: in a pooled meta-analysis of 48 biopsy series, isolated diabetic nephropathy accounted for half of diagnoses (50%), non-diabetic renal disease for 37%, and mixed lesions combining diabetic and non-diabetic pathology for 20% [26]. Mixed disease carries direct prognostic and therapeutic implications [27], yet regional series rarely report it separately [7-25]. Therefore, we defined an additional “DKD+” category for diabetic kidney disease mixed with non-diabetic pathology.

To address this gap, we conducted a single-center study of biopsy-proven GN at King Fahad Armed Forces Hospital, Jeddah, over 24 years (2000-2024), with two primary aims: to characterize the clinicopathological spectrum and to identify predictors of renal function decline at one year among patients undergoing native kidney biopsy. Secondary aims were to describe, as an exploratory analysis, patients with diabetic kidney disease and a coexisting non-diabetic lesion (DKD+) and to compare these findings descriptively with four decades of Saudi biopsy literature.

## Materials and methods

Study design and setting

We conducted a retrospective study at the Renal Diseases and Transplantation Center, King Fahad Armed Forces Hospital, Jeddah, involving all native kidney biopsy reports issued between January 2000 and December 2024. No retrievable records existed before 2000. Native kidney biopsies were performed at the discretion of the treating nephrologist for standard indications, including nephrotic syndrome, nephrotic-range proteinuria, unexplained acute or chronic kidney injury, and hematuria. In patients with diabetes, biopsy was performed when atypical features suggested non-diabetic disease, such as absent retinopathy, rapid eGFR decline, or active urinary sediment.

Study population

We included native kidney biopsies from patients older than 14 years with enough tissue for diagnosis. Of 700 biopsies screened, 367 were excluded, leaving 333 in the study cohort (Figure 1).

**Figure 1:**
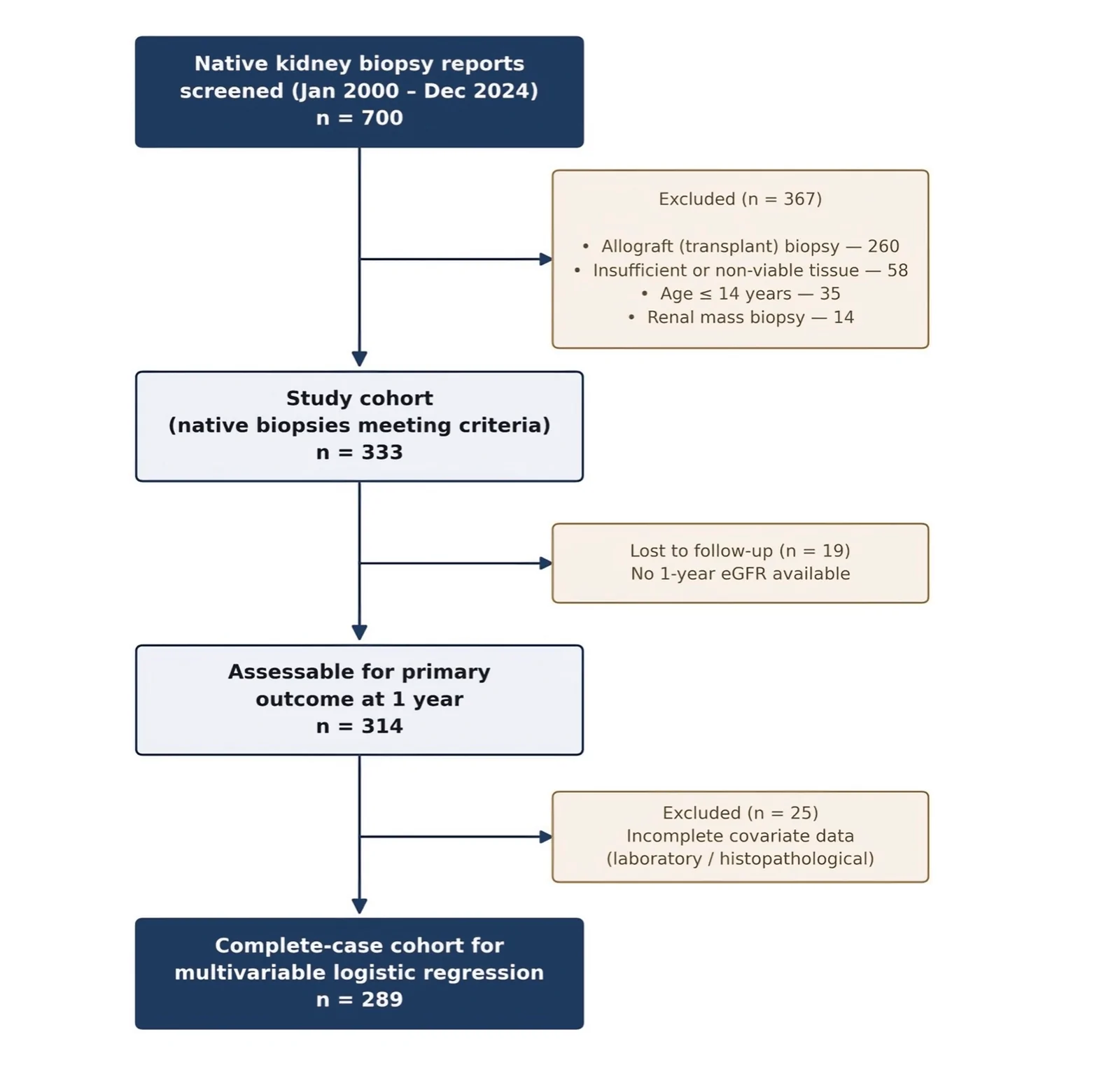
STROBE flow diagram of biopsy screening, inclusion, follow-up, and the complete case cohort used for multivariable logistic regression

Data collection

We collected demographic details, comorbid conditions, laboratory findings, as well as histopathological findings from electronic medical records and pathology documentation.

Baseline time was defined as the biopsy date. eGFR was calculated from serum creatinine using the 2021 CKD-EPI (Chronic Kidney Disease Epidemiology Collaboration) equation [28]. All biopsies were examined by light microscopy with immunofluorescence; electron microscopy was performed in selected cases. Chronic damage was recorded as the percentage of globally sclerosed glomeruli, interstitial fibrosis and tubular atrophy (IFTA), and arteriosclerosis, each graded by percentage of cortex involvement as none, mild (50%). Diagnoses followed the original pathology report issued at the time of biopsy; slides were not centrally re-reviewed. We added one category, DKD+, for biopsies showing diabetic kidney disease (diffuse or nodular mesangial expansion and/or glomerular basement membrane thickening) together with non-diabetic pathology.

Outcomes and statistical analysis

The primary outcome was a composite of a ≥40% decline in eGFR from baseline or an eGFR below 15 mL/min/1.73 m² at one year. We used the 40% threshold based on the NKF-FDA workshop [29], with a ≥30% decline used in sensitivity analyses. One-year eGFR was calculated from the serum creatinine value closest to 12 months after biopsy, within a window of 10-14 months.

Continuous variables were summarized as median and interquartile range, and categorical variables as counts and percentages (N (%)). Groups were compared using the Kruskal-Wallis test and Fisher’s exact test. A two-sided p < 0.05 was considered statistically significant. Predictors of the primary outcome were assessed by multivariable logistic regression. Patients lost to follow-up (n = 19) were excluded, leaving 314 assessable for the outcome; after excluding 25 with incomplete covariate data, models were fitted on 289. Analyses were done in R version 4.3.1 (R Foundation for Statistical Computing, Vienna, Austria).

For the comparison with published Saudi series, we searched PubMed, Scopus, and Google Scholar for Saudi studies reporting biopsy-proven glomerular disease, using the terms “glomerulonephritis” OR “kidney biopsy” AND “Saudi Arabia.” We included original series and registries and excluded review articles, with no date restriction. Studies were grouped into four eras for comparison over time. Comparison was as narrative context rather than a systematic review. 

Ethics

The Research Ethics Committee of Armed Forces Hospital, Jeddah, Saudi Arabia approved the study (approval number REC953). The study was conducted in accordance with the Declaration of Helsinki.

## Results

In this cohort, the median age was 38 years (IQR 28-51), and 173 patients (52%) were female. Hypertension was present in 239 (72%) and diabetes in 104 (31%). At biopsy time, most patients had reduced kidney function, with a median eGFR of 37 mL/min/1.73 m² (IQR 15-80), heavy proteinuria (median 2.8 g/day, IQR 1.4-6.0), and hypoalbuminemia (median 30 g/L, IQR 23-36). Chronic histological damage was common, with moderate to severe IFTA in 86 biopsies (26%) and moderate to severe arteriosclerosis in 45 (13%) (Table 1).

**Table 1:** Baseline demographic, clinical, laboratory, and histopathological characteristics of the study cohort Continuous variables are presented as median (IQR). Categorical variables are presented as N (%). Medication data were available for 289 patients, which is the denominator for medication percentages. A hyphen (-) marks category headings with no value. eGFR, estimated glomerular filtration rate; HbA1c, glycated hemoglobin; IFTA, interstitial fibrosis and tubular atrophy; RAS, renin-angiotensin system; SGLT2, sodium-glucose co-transporter 2

Characteristic	Overall (N = 333)
Demographics	
Age (years)	38 (28-51)
Age Group (years)	
< 20.00	23 (6.9%)
20.00 - 39.00	155 (47%)
40.00 - 59.00	102 (31%)
≥ 60.00	53 (16%)
Gender	
Female	173 (52%)
Male	160 (48%)
Clinical Characteristics	
Hypertension (HTN)	239 (72%)
Diabetes Mellitus (DM)	104 (31%)
Baseline Creatinine (µmol/L)	170 (91-370)
Baseline eGFR (mL/min/1.73m²)	37 (15-80)
Laboratory Findings	
HbA1c (%)	5.5 (5.1-6.5)
24-Hour Protein (g/day)	2.8 (1.4-6.0)
Albumin (g/L)	30 (23-36)
Total Protein (g/L)	59 (50-66)
Hemoglobin (g/dL)	10.7 (9.4-12.7)
Calcium (mmol/L)	2.2 (2.0-2.3)
Pathological & Biopsy Findings	
Global Sclerosis Group	
No Sclerosis	88 (26%)
< 25%	121 (36%)
25 - 50%	79 (24%)
> 50%	45 (14%)
Segmental Sclerosis, present	182 (55%)
Interstitial Fibrosis and Tubular Atrophy (IFTA)	
No	90 (27%)
Mild (< 25%)	157 (47%)
Moderate (25 - 50%)	66 (20%)
Severe (> 50%)	20 (6.0%)
Arteriosclerosis	
No	215 (65%)
Mild (< 25%)	73 (22%)
Moderate (25 - 50%)	33 (9.9%)
Severe (> 50%)	12 (3.6%)
Medications (N=289)	
RAS Inhibitor Use	
No	16 (5.5%)
Yes	273 (94%)
SGLT2 Inhibitor Use	
No	161 (56%)
Yes	128 (44%)

Table 2 shows the distribution of biopsy-proven glomerular diseases. The most common diagnoses were lupus nephritis (72, 21.6%), followed by FSGS (60, 18%), diabetic nephropathy (46, 13.8%), and IgA nephropathy (35, 10.5%). Other frequent disorders include interstitial nephritis, membranous nephropathy, and membranoproliferative glomerulonephritis (MPGN). The identification of minimal change disease (MCD) and thrombotic microangiopathy (TMA) was rare, but they also showed consistent results for a diagnosis of diabetic kidney disease with mixed pathology.

**Table 2:** Spectrum of biopsy-proven glomerular disease Data are presented as N(%), where N is the number of biopsies and (%) is the percentage of the whole cohort (N=333). DKD+: diabetic kidney disease mixed with non-diabetic pathology; FSGS: focal segmental glomerulosclerosis; MPGN: membranoproliferative glomerulonephritis; TMA: thrombotic microangiopathy; MGRS: monoclonal gammopathy of renal significance

Diagnosis	N (%)
Lupus nephritis	72 (21.6%)
FSGS	60 (18%)
Diabetic nephropathy	46 (13.8%)
IgA nephropathy	35 (10.5%)
Interstitial nephritis	26 (7.8%)
DKD +	24 (7.2%)
Membranous nephropathy	23 (6.9%)
MPGN	18 (5.4%)
Minimal change disease	13 (3.9%)
TMA	10 (3%)
MGRS/paraproteinemia	6 (1.8%)
Total	333

Characteristics by pathological diagnosis are shown in Table 3. Patients with DKD+ were the oldest (median 57 years), while those with MCD and TMA were the youngest (both 27 years). Female predominance was most marked in lupus nephritis (61, 85%), while male predominance was greatest in DKD+ (17, 71%), diabetic nephropathy (30, 65%), and interstitial nephritis (17, 65%). Hypertension was present in almost all patients with diabetic nephropathy (43, 93%) and DKD+ (22, 92%) but was absent in MCD, and comorbid diabetes clustered as expected in diabetic nephropathy (46, 100%) and DKD+ (23, 96%). Baseline kidney function showed a broad range: median eGFR was lowest in DKD+ (8 mL/min/1.73 m²) and interstitial nephropathy (11) and best preserved in MCD (119) and FSGS (53). Proteinuria was heaviest in MCD (median 7.3 g/day) and membranous nephropathy (6.7), consistent with nephrotic presentation, and lowest in interstitial nephropathy (0.5); serum albumin was lowest in MCD (18 g/L). HbA1c was highest in the diabetic groups (7.3% and 7.2%).

**Table 3:** Demographic, clinical, and laboratory characteristics by pathological diagnosis Continuous variables are presented as median (IQR), and categorical variables are presented as N (%). Composite outcome percentages are calculated among patients with one-year follow-up. p-values for continuous variables were calculated using the Kruskal-Wallis rank sum test. p-values for categorical variables were calculated using Fisher's exact test with simulated p-values (based on 2,000 replicates). p < 0.05 was considered statistically significant; * denotes a statistically significant p-value. A hyphen indicates no value. Diabetes refers to a documented clinical diagnosis before biopsy. Discrepancies in sums for 'Overall' reflect missing data for certain variables in the raw dataset. DN: diabetic nephropathy; DKD+: diabetic kidney disease mixed with non-diabetic pathology; Int. Neph: interstitial nephritis; Mem.: membranous nephropathy; MCD: minimal change disease; MGRS: monoclonal gammopathy of renal significance; MPGN: membranoproliferative glomerulonephritis; PCR: protein-creatinine ratio; TMA: thrombotic microangiopathy; RAS: renin-angiotensin system; SGLT2: sodium-glucose co-transporter 2; IFTA: interstitial fibrosis and tubular atrophy

Characteristic Demographics	Overall (N=333)	Lupus (N=72)	FSGS (N=60)	DN (N=46)	IgA (N=35)	Int. Neph. (N=26)	DKD+ (N=24)	Mem. (N=23)	MPGN (N=18)	MCD (N=13)	TMA (N=10)	MGRS/Paraproteinemia (N=6)	p-value
Age (years)	38 (28, 51)	31 (26, 37)	35 (28, 43)	51 (44, 61)	38 (24, 45)	46 (32, 65)	57 (48, 70)	38 (34, 53)	43 (33, 60)	27 (18, 31)	27 (26, 33)	45 (40, 60)	<0.001*
Age Group (years)													<0.001*
< 20.00	23 (6.9%)	9 (13%)	5 (8.3%)	0 (0%)	2 (5.7%)	2 (7.7%)	0 (0%)	0 (0%)	1 (5.6%)	4 (31%)	0 (0%)	0 (0%)	-
20.00 - 39.00	155 (47%)	48 (67%)	36 (60%)	9 (20%)	19 (54%)	6 (23%)	3 (13%)	12 (52%)	6 (33%)	6 (46%)	9 (90%)	1 (17%)	-
40.00 - 59.00	102 (31%)	13 (18%)	14 (23%)	25 (54%)	11 (31%)	10 (38%)	10 (42%)	7 (30%)	6 (33%)	2 (15%)	1 (10%)	3 (50%)	-
≥ 60.00	53 (16%)	2 (2.8%)	5 (8.3%)	12 (26%)	3 (8.6%)	8 (31%)	11 (46%)	4 (17%)	5 (28%)	1 (7.7%)	0 (0%)	2 (33%)	-
Gender													<0.001*
Female	173 (52%)	61 (85%)	32 (53%)	16 (35%)	17 (49%)	9 (35%)	7 (29%)	10 (43%)	7 (39%)	6 (46%)	5 (50%)	3 (50%)	-
Male	160 (48%)	11 (15%)	28 (47%)	30 (65%)	18 (51%)	17 (65%)	17 (71%)	13 (57%)	11 (61%)	7 (54%)	5 (50%)	3 (50%)	-
Clinical Characteristics													
Hypertension (HTN)													<0.001*
No	94 (28%)	10 (14%)	25 (42%)	3 (6.5%)	10 (29%)	7 (27%)	2 (8.3%)	14 (61%)	4 (22%)	13 (100%)	2 (20%)	4 (67%)	-
Yes	239 (72%)	62 (86%)	35 (58%)	43 (93%)	25 (71%)	19 (73%)	22 (92%)	9 (39%)	14 (78%)	0 (0%)	8 (80%)	2 (33%)	-
Diabetes Mellitus (DM)													<0.001*
No	229 (69%)	69 (96%)	51 (85%)	0 (0%)	32 (91%)	17 (65%)	1 (4.2%)	19 (83%)	13 (72%)	12 (92%)	10 (100%)	5 (83%)	-
Yes	104 (31%)	3 (4.2%)	9 (15%)	46 (100%)	3 (8.6%)	9 (35%)	23 (96%)	4 (17%)	5 (28%)	1 (7.7%)	0 (0%)	1 (17%)	-
Baseline Creatinine (µmol/L)	170 (91, 370)	93 (56, 187)	138 (79, 231)	284 (162, 538)	205 (131, 295)	534 (288, 703)	590 (164, 762)	103 (58, 168)	228 (130, 571)	68 (50, 155)	324 (168, 421)	344 (138, 456)	<0.001*
Baseline eGFR (mL/min/1.73m²)	37 (15, 80)	75 (37, 117)	53 (26, 103)	19 (10, 38)	33 (18, 73)	11 (7, 23)	8 (6, 39)	75 (36, 110)	27 (9, 40)	119 (54, 130)	20 (15, 40)	19 (10, 50)	<0.001*
Composite Outcome													<0.001*
No	190 (61%)	53 (75%)	40 (69%)	10 (22%)	23 (68%)	14 (64%)	11 (52%)	14 (70%)	10 (63%)	10 (83%)	5 (50%)	0 (0%)	-
Yes	124 (39%)	18 (25%)	18 (31%)	36 (78%)	11 (32%)	8 (36%)	10 (48%)	6 (30%)	6 (38%)	2 (17%)	5 (50%)	4 (100%)	-
Laboratory Findings													
HbA1c (%)	5.50 (5.10, 6.50)	5.20 (5.10, 5.40)	5.45 (4.90, 5.70)	7.30 (6.20, 9.00)	5.35 (4.95, 5.70)	6.30 (5.30, 7.80)	7.20 (6.50, 7.80)	5.40 (5.20, 6.20)	5.50 (5.15, 7.10)	5.30 (5.05, 5.90)	4.30 (4.10, 4.70)	3.60 (2.00, 4.50)	<0.001*
PCR (g/g)	2.4 (1.3, 4.9)	2.3 (1.0, 3.4)	2.2 (1.4, 4.0)	5.6 (1.8, 9.6)	1.9 (0.7, 2.7)	0.5 (0.2, 1.9)	3.0 (1.2, 7.7)	4.1 (2.6, 6.9)	2.0 (1.0, 3.6)	3.0 (2.5, 6.8)	3.3 (1.7, 3.5)	4.9 (2.8, 5.6)	<0.001*
24-Hour urine Protein (g/day)	2.8 (1.4, 6.0)	2.4 (1.5, 4.5)	2.9 (2.2, 6.0)	4.5 (1.6, 9.0)	1.8 (0.8, 2.9)	0.5 (0.3, 1.5)	3.4 (1.0, 8.0)	6.7 (5.0, 11.0)	1.9 (1.1, 3.2)	7.3 (4.3, 8.0)	1.7 (1.1, 4.9)	5.7 (2.5, 12.0)	<0.001*
Albumin (g/L)	30 (23, 36)	25 (21, 33)	34 (24, 37)	30 (26, 33)	35 (30, 39)	34 (29, 39)	28 (22, 36)	25 (18, 31)	33 (30, 39)	18 (16, 23)	28 (26, 36)	33 (23, 41)	<0.001*
Hemoglobin (g/dL)	10.75 (9.40, 12.70)	10.20 (9.07, 11.55)	12.45 (10.70, 14.25)	10.00 (9.20, 11.00)	11.05 (10.15, 13.10)	10.50 (9.30, 12.10)	10.45 (8.83, 11.50)	12.40 (10.60, 13.90)	10.35 (9.60, 11.40)	13.00 (12.20, 14.70)	9.60 (9.00, 10.00)	8.15 (6.80, 10.50)	<0.001*
Calcium (mmol/L)	2.20 (2.01, 2.30)	2.01 (1.84, 2.23)	2.30 (2.20, 2.30)	2.06 (1.95, 2.17)	2.20 (2.09, 2.30)	2.20 (2.10, 2.30)	2.20 (1.91, 2.29)	2.20 (2.10, 2.30)	2.20 (2.10, 2.26)	2.35 (2.30, 2.40)	2.14 (2.00, 2.29)	2.17 (2.02, 2.27)	<0.001*
Medications													
RAS Inhibitor Use													<0.001*
No	16 (5.5%)	4 (14%)	0 (0%)	0 (0%)	12 (34%)	0 (0%)	0 (0%)	0 (0%)	0 (0%)	0 (0%)	0 (0%)	0 (0%)	-
Yes	273 (94%)	25 (86%)	60 (100%)	46 (100%)	23 (66%)	26 (100%)	24 (100%)	23 (100%)	17 (100%)	13 (100%)	10 (100%)	6 (100%)	-
SGLT2 Inhibitor Use													<0.001*
No	161 (56%)	29 (100%)	20 (33%)	37 (80%)	31 (89%)	6 (23%)	10 (42%)	7 (30%)	16 (94%)	1 (7.7%)	1 (10%)	3 (50%)	-
Yes	128 (44%)	0 (0%)	40 (67%)	9 (20%)	4 (11%)	20 (77%)	14 (58%)	16 (70%)	1 (5.9%)	12 (92%)	9 (90%)	3 (50%)	-

Of the 314 patients with follow-up, 124 (39%) met the primary outcome (a ≥ 40% decline in eGFR from baseline or an eGFR < 15 mL/min/1.73 m² at one year). Outcome rates varied by diagnosis (p < 0.001), being highest in diabetic nephropathy (36, 78%) and DKD+ (10, 48%) and lowest in MCD (2, 17%), lupus nephritis (18, 25%), and membranous nephropathy (6, 30%). Rates in the smallest diagnostic groups should be interpreted with caution, as a single patient shifts the proportion by several percentage points.

Medication data were available for 289 patients after excluding 19 lost to follow-up and 25 with missing data. Renin-angiotensin system (RAS) inhibitors were used in almost all patients (273, 94%), while sodium-glucose cotransporter 2 (SGLT2) inhibitor use was lower and more variable across diagnoses (128, 44%).

The distribution of histopathological features varied significantly across the eleven diagnoses (Table 4). Global glomerulosclerosis was present in most biopsies overall (245, 74%), with the greatest burden (>50% of glomeruli) seen in diabetic nephropathy (14, 30%), IgA nephropathy (10, 29%), and MPGN (5, 28%). In contrast, MCD and monoclonal gammopathy of renal significance (MGRS)/paraproteinemia showed little or no global sclerosis (p < 0.001).

**Table 4:** Histopathological findings by pathological diagnosis Data are presented as N (%). p-values were calculated using Fisher’s exact test with simulated p-values (based on 2,000 replicates). p < 0.05 was considered statistically significant; * denotes a statistically significant p-value. A hyphen (-) indicates no value. DN: diabetic nephropathy; DKD+: diabetic kidney disease mixed with non-diabetic pathology; Int. Neph: interstitial nephritis; Mem.: membranous nephropathy; MCD: minimal change disease; MGRS: monoclonal gammopathy of renal significance; MPGN: membranoproliferative glomerulonephritis; PCR: protein-creatinine ratio; TMA: thrombotic microangiopathy; RAS: renin-angiotensin system; SGLT2: sodium-glucose co-transporter 2; IFTA: interstitial fibrosis and tubular atrophy

Characteristic	Overall (N=333)	MGRS (N=6)	DKD+ (N=24 )	DN (N=46)	FSGS (N=60 )	IgA (N=35)	Int. Neph. (N=26)	Lupus (N=72)	MCD (N=13)	Mem. (N=23 )	MPGN (N=18)	TMA (N=10)	pvalue
Global Sclerosis Group													<0.001*
No sclerosis	88 (26%)	2 (33%)	5 (21%)	5 (11%)	9 (15%)	5 (14%)	12 (46%)	20 (28%)	9 (69%)	11 (48%)	6 (33%)	4 (40%)	-
< 25%	121 (36%)	4 (67%)	9 (38%)	13 (28%)	21 (35%)	11 (31%)	6 (23%)	35 (49%)	4 (31%)	8 (35%)	4 (22%)	6 (60%)	-
25 - 50%	79 (24%)	0 (0%)	7 (29%)	14 (30%)	22 (37%)	9 (26%)	6 (23%)	15 (21%)	0 (0%)	3 (13%)	3 (17%)	0 (0%)	-
> 50%	45 (14%)	0 (0%)	3 (13%)	14 (30%)	8 (13%)	10 (29%)	2 (7.7%)	2 (2.8%)	0 (0%)	1 (4.3%)	5 (28%)	0 (0%)	
Segmental Sclerosis Group													<0.00 1*
No sclerosis	151 (45%)	5 (83%)	16 (67%)	18 (39%)	10 (17%)	6 (17%)	23 (88%)	28 (39%)	13 (100%)	12 (52%)	14 (78%)	6 (60%)	-
< 25%	125 (38%)	1 (17%)	5 (21%)	16 (35%)	36 (60%)	23 (66%)	0 (0%)	29 (40%)	0 (0%)	9 (39%)	2 (11%)	4 (40%)	-
25 - 50%	49 (15%)	0 (0%)	3 (13%)	10 (22%)	11 (18%)	6 (17%)	3 (12%)	14 (19%)	0 (0%)	1 (4.3%)	1 (5.6%)	0 (0%)	-
> 50%	8 (2.4%)	0 (0%)	0 (0%)	2 (4.3%)	3 (5.0%)	0 (0%)	0 (0%)	1 (1.4%)	0 (0%)	1 (4.3%)	1 (5.6%)	0 (0%)	-
IFTA													<0.00 1*
No	90 (27%)	3 (50%)	7 (29%)	4 (8.7%)	15 (25%)	4 (11%)	6 (23%)	32 (44%)	9 (69%)	3 (13%)	5 (28%)	2 (20%)	-
Mild (< 25%)	157 (47%)	2 (33%)	14 (58%)	16 (35%)	27 (45%)	25 (71%)	14 (54%)	29 (40%)	4 (31%)	15 (65%)	5 (28%)	6 (60%)	-
Moderate (25 - 50%)	66 (20%)	0 (0%)	2 (8.3%)	15 (33%)	16 (27%)	5 (14%)	4 (15%)	10 (14%)	0 (0%)	4 (17%)	8 (44%)	2 (20%)	-
Severe (> 50%)	20 (6.0%)	1 (17%)	1 (4.2%)	11 (24%)	2 (3.3%)	1 (2.9%)	2 (7.7%)	1 (1.4%)	0 (0%)	1 (4.3%)	0 (0%)	0 (0%)	-
Arteriosclerosis													0.015*
No	215 (65%)	3 (50%)	14 (58%)	25 (54%)	37 (62%)	24 (69%)	15 (58%)	55 (76%)	13 (100%)	16 (70%)	9 (50%)	4 (40%)	-
Mild (< 25%)	73 (22%)	2 (33%)	7 (29%)	7 (15%)	13 (22%)	8 (23%)	9 (35%)	13 (18%)	0 (0%)	6 (26%)	3 (17%)	5 (50%)	-
Moderate (25 - 50%)	33 (9.9%)	1 (17%)	2 (8.3%)	10 (22%)	7 (12%)	2 (5.7%)	2 (7.7%)	2 (2.8%)	0 (0%)	1 (4.3%)	6 (33%)	0 (0%)	-
Severe (> 50%)	12 (3.6%)	0 (0%)	1 (4.2%)	4 (8.7%)	3 (5.0%)	1 (2.9%)	0 (0%)	2 (2.8%)	0 (0%)	0 (0%)	0 (0%)	1 (10%)	-

Segmental sclerosis was, as expected, most characteristic of FSGS (50, 83%) and IgA nephropathy (29, 83%), while it was absent in MCD and uncommon in interstitial nephritis (none in 23 (88%)). IFTA was present in 243 (73%) biopsies overall and was most severe in DN, where 11 (24%) showed severe (>50%) involvement, consistent with chronic diabetic injury. Conversely, MCD showed no moderate to severe fibrosis. Arteriosclerosis was less strongly associated with diagnosis (p = 0.015) but followed expected vascular-risk patterns, with moderate to severe arteriosclerosis most frequent in MPGN (6, 33%) and DN (14, 30%) and absent in MCD.

In the multivariable logistic regression (Table 5), chronic histopathological damage was among the strongest independent predictors of the primary composite outcome. Moderate-to-severe IFTA more than doubled the odds of reaching the endpoint (aOR 2.13, 95% CI 1.14-3.98; p = 0.018), as did moderate-to-severe arteriosclerosis (aOR 2.38, 95% CI 1.09-5.26; p = 0.034), with both associations independent of diagnosis, baseline kidney function, and proteinuria. Among clinical variables, higher 24-hour urinary protein (aOR 1.08 per g/day, 95% CI 1.01-1.15; p = 0.026) and lower baseline eGFR (aOR 0.98 per mL/min/1.73 m², 95% CI 0.97-0.99; p < 0.001) were independently associated with the outcome, while age and sex were not. Neither RAS inhibitor nor SGLT2 inhibitor use was significantly associated with the primary outcome. Because medication status reflected post-biopsy prescribing rather than randomized exposure, these estimates are susceptible to confounding by indication and should not be interpreted causally. The model was fitted in 289 patients with complete data.

**Table 5:** Logistic regression of predictors of the primary composite outcome (≥40% eGFR decline or eGFR < 15 mL/min/1.73 m² at 1 year), with sensitivity analyses using a ≥30% eGFR decline threshold Odds ratios for continuous variables are per 1-unit increase (eGFR, mL/min/1.73 m²; 24-hour urine protein, g/day). The model was fitted in 289 patients with complete data. p < 0.05 was considered statistically significant; * denotes a statistically significant p-value. aOR, adjusted odds ratio; CI, confidence interval; IFTA, interstitial fibrosis and tubular atrophy; eGFR, estimated glomerular filtration rate; RAS, renin-angiotensin system; SGLT2, sodium-glucose co-transporter 2

	Primary: ≥40% eGFR decline or eGFR < 15		Sensitivity: composite (30% decline or eGFR < 15)		Sensitivity: 30% eGFR decline	
Predictor	aOR (95% CI)	p-value	aOR (95% CI)	p-value	aOR (95% CI)	p-value
Age (years)	0.99 (0.98 - 1.01)	0.500	1.00 (0.99 - 1.02)	0.600	0.99 (0.97 - 1.01)	0.300
Gender						
Female	Reference	-	Reference	-	Reference	-
Male	0.59 (0.33 - 1.05)	0.071	0.68 (0.38 - 1.20)	0.200	0.75 (0.40 - 1.40)	0.400
Baseline eGFR	0.98 (0.97 - 0.99)	<0.001*	0.99 (0.98 - 1.00)	0.002*	1.00 (0.99 - 1.01)	>0.900
24-Hour urine Protein	1.08 (1.01 - 1.15)	0.026*	1.09 (1.03 - 1.17)	0.006*	1.14 (1.06 - 1.22)	<0.001*
IFTA						
None/Mild	Reference	-	Reference	-	Reference	-
Moderate/Severe	2.13 (1.14 - 3.98)	0.018*	3.02 (1.61 - 5.78)	<0.001*	2.74 (1.36 - 5.59)	0.005*
Arteriosclerosis						
No/Mild	Reference	-	Reference	-	Reference	-
Moderate/Severe	2.38 (1.09 - 5.26)	0.034*	2.50 (1.11 - 5.88)	0.031*	1.37 (0.58 - 3.13)	0.500
RAS Inhibitor Use						
No	Reference	-	Reference	-	Reference	-
Yes	0.68 (0.21 - 2.26)	0.500	0.88 (0.27 - 3.09)	0.800	1.22 (0.34 - 5.80)	0.800
SGLT2 Inhibitor Use						
No	Reference	-	Reference	-	Reference	-
Yes	0.67 (0.38 - 1.18)	0.200	0.64 (0.36 - 1.12)	0.120	0.50 (0.26 - 0.94)	0.035*

Associations were also observed across different outcome definitions. In sensitivity analyses using a a 30% decline threshold, IFTA and proteinuria remained significant across all definitions, and IFTA showed a consistent, graded effect (aOR 3.02 for the composite outcome of a 30% decline in eGFR or eGFR < 15 mL/min/1.73 m² and 2.74 for a 30% decline alone; both p < 0.005). Arteriosclerosis remained significant in the composite-outcome analyses, but its association was no longer statistically significant when the outcome was restricted to a 30% decline alone (aOR 1.37; p = 0.500), indicating that its predictive contribution was strongest for the more severe composite endpoints.

## Discussion

In this 24-year single-center cohort of 333 biopsy-proven glomerular diseases, lupus nephritis was the most common diagnosis overall and FSGS was the most common primary glomerular lesion. Of the 314 patients with follow-up at one year, 124 (39%) reached the composite renal endpoint. Our principal prognostic findings are that the severity of chronic histological damage, especially IFTA, and arteriosclerosis independently predicted functional decline at one year, alongside heavy proteinuria and lower baseline eGFR. Age and sex were not predictive, and post-biopsy medication use was not associated with the primary outcome.

The independent association between chronic histological damage and functional decline is clinically relevant. IFTA and arteriosclerosis represent an irreversible burden of injury within the kidney, and both doubled the odds of the composite outcome after adjustment, suggesting that they reflect risk that the diagnosis alone cannot capture. Two patients with the same glomerular disease but different degrees of tubulointerstitial and vascular scarring may therefore follow different courses, with chronicity acting as a final common endpoint for glomerular injury of any cause. This is consistent with the long-standing observation that the degree of tubulointerstitial damage, rather than the glomerular lesion in isolation, is among the strongest histological correlates of renal prognosis [30]. The concurrent contributions of proteinuria and baseline eGFR match their recognized roles as markers of progression [31], and their retention alongside the chronicity indices shows that histology adds prognostic value beyond routine clinical measures.

A distinctive feature of our cohort was the DKD+ group, in which biopsy showed non-diabetic pathology alongside diabetic changes. These patients had the lowest baseline eGFR in the series (median 8 mL/min/1.73 m²) and reached the composite outcome in 10 (48%) cases, second only to isolated diabetic nephropathy. This reinforces a point of clinical importance: kidney disease in patients with diabetes cannot be assumed to be diabetic nephropathy. In a pooled analysis of 48 biopsy studies, isolated diabetic nephropathy accounted for only about half of diagnoses (50%), with 37% showing non-diabetic disease and 20% showing mixed lesions [26]. Our data extend these findings by linking mixed pathology to worse short-term kidney function. In practice, atypical features such as absent retinopathy, rapid loss of function, or sudden heavy proteinuria should lower the threshold for biopsy, since finding a second, treatable lesion may change both prognosis and treatment.

Our findings were consistent with four decades of Saudi biopsy literature (Table 6) [7-25]. FSGS has been the most consistently reported primary GN, leading in most series from the earliest studies [7-9] through to modern multicenter analyses [22,23]. The first Saudi series, reported in 1984, already found a high frequency of FSGS, with comparatively low frequencies of membranous nephropathy and amyloidosis [7], a pattern that persisted, particularly in men and young adults [13,20], and was later associated with obesity and metabolic factors [16]. Mesangial proliferative GN and IgA nephropathy were reported as the second most common glomerular lesions, while MCD, though less frequent, was repeatedly associated with the most favorable outcomes. Two regional exceptions stand out: the southern series from Abha was the only one in which MPGN predominated [10], and series from the western region (Jeddah) alone reported membranous nephropathy as the leading primary lesion [17,24]. These geographically clustered findings suggest genuine regional variation rather than coincidence, a signal supported by variation observed between centers within the unified-protocol multicenter study [23].

**Table 6:** Four decades of published Saudi kidney biopsy literature Studies are listed in order of publication year and categorized into four eras, reflecting the development of nephropathology in Saudi Arabia. Era boundaries refer to publication year, not the period of biopsy collection. Biopsies = number of biopsies (N) in each study; numbers in [..] are reference numbers. The most common GN is presented in descending order of frequency, as reported in each study; semicolons separate primary and secondary GN according to how the original papers presented them. Percentages are not shown because methods and denominators differed between studies; therefore, no pooled comparison should be inferred, and no assumption of meta-analytic equivalence should be made. AFH, Armed Forces Hospital; DN, diabetic nephropathy; EM, electron microscopy; FSGS, focal segmental glomerulosclerosis; GN, glomerulonephritis; IF, immunofluorescence; IgA/IgAN, IgA nephropathy; KAU, King Abdulaziz University; KFMC, King Fahd Medical City; KKUH, King Khalid University Hospital; LM, light microscopy; LN, lupus nephritis; MCD, minimal change disease; Membranous GN, membranous glomerulonephritis; MPGN, membranoproliferative (mesangiocapillary) glomerulonephritis; MsPGN, mesangial proliferative glomerulonephritis; NR, not reported; retro, retrospective; SCN, sickle cell nephropathy; SLE, systemic lupus erythematosus

#	Author/Year	Center/Design	Period	Biopsies	Most common GN	Study-specific notes
Era 1 - Foundational retrospective studies (1984-1999)						
1	Qunibi et al. (1984) [7]	Single (KFSH), retro	1975-1983	147	FSGS	Earliest Saudi biopsy series
2	Akhtar et al. (1990) [8]	Multicenter, retro	1983-1988	380	FSGS, MsPGN	Selection/information bias; MPGN type 2 absent
3	Mitwalli et al. (1996) [9]	Single (KKUH), retro	1989-1994	186	FSGS, MsPGN; LN	Highest FSGS reported; MCD very low (1.4%)
4	Al-Homrany (1999) [10]	Single (Abha), retro	1989-1994	166	MPGN, IgA; LN	Only series with MPGN predominant
Era 2 - First registry and prospective work (2000)						
5	Huraib et al. (2000) [11]	Multicenter registry, retro	NR	1294	FSGS, MPGN; LN	Largest series; amyloidosis low (3.2%)
6	Mousa et al. (2000) [12]	Single(AFH), prospective	1999	52	FSGS, IgA; LN	First prospective series; high IgA; MPGN only 2%
7	Mitwalli et al. (2000) [13]	Single (KKUH), retro	1994-1999	200	FSGS, MsPGN; LN	FSGS predominant in males, young adults
8	Bernieh et al. (2000) [14]	Single (Madinah), retro	1990-1999	85	MCD, MsPGN, FSGS; LN	LM only (no IF/EM);MCD best
Era 3 - Regional single-center expansion (2004-2015)						
9	Al Wakeel et al. (2004) [15]	Single (KKUH), retro	1990-2001	120	FSGS, IgA, MGN	Outcome study; FSGS worst, MCD best
10	Alkhunaizi (2007) [16]	Single (Dhahran), prospective	1998-2005	100	FSGS, IgA; LN, SCN	FSGS-Obesity link; Sickle-cell nephropathy notable
11	Jalalah (2009) [17]	Single (Western), retro	1989-2007	296	MGN, FSGS, IgA	Only Saudi series with MGN predominant
12	Shawarby et al. (2010) [18]	Single (AL-Khobar), retro	1986-2008	233	MCD, MsPGN, FSGS; LN	131 cases overlap Huraib 2000 registry
13	Saleemi et al. (2012) [19]	Single (AlQassim), retro	2005-2010	126	LN; FSGS, MCD	IF only after 2007; no EM
14	Nawaz et al. (2013) [20]	Single (AFH), retro	2005-2009	348	FSGS, MCD, MPGN; LN	Marked male predominance across subtypes
Era 4 - Modern multicenter analysis and trend detection (2016-2022)						
15	AlMatham et al. (2017) [21]	Single (KFMC), retro	2007-2016	166	FSGS, MCD, MGN	SLE-associated GN analyzed separately
16	AlFaadhel et al. (2019) [22]	Multicenter, retro	1998-2017	1070	FSGS, IgA, MGN; LN, DN	DN rose 1.4% to 10.2% across eras
17	Al-Homrany et al. (2019) [23]	Multicenter, prospective	2010-2012	405	FSGS, IgA, MsPGN, MPGN; LN	Unified protocol; regional variation noted
18	Jalalah (2020) [24]	Single (Western), retro	1988-2013	711	MGN, FSGS; LN	Two-period trend analysis; extends Jalalah (2009)
19	Albeladi et al. (2022) [25]	Single (KAU), retro	2010-2018	55	FSGS; LN	Small single-center series

Across secondary GN, lupus nephritis was dominant throughout all decades, reflecting the burden of systemic lupus erythematosus in the Kingdom, while amyloidosis remained uncommon even in the largest registry [11]. The clearest temporal trend was the rise of diabetic nephropathy, which increased from 1.4% to 10.2% across successive eras in the national multicenter analysis [22], reflecting the rise in diabetes prevalence and reshaping the secondary GN landscape in the modern period (Figure 2). The same trend was visible in our own cohort, with its substantial diabetic and DKD+ representation. This pattern should be interpreted descriptively, as the series differed in design, study population, and diagnostic completeness. Several early and peripheral centers relied on light microscopy alone, and the literature is dominated by single-center retrospective studies concentrated in the central region. In addition, partial overlap between some cohorts prevents pooled quantification. Against this background, the Saudi literature has been descriptive, and although one earlier series reported outcomes by diagnosis [15], multivariable analyses linking biopsy findings to renal function have been lacking. Our study addresses that gap.

**Figure 2:**
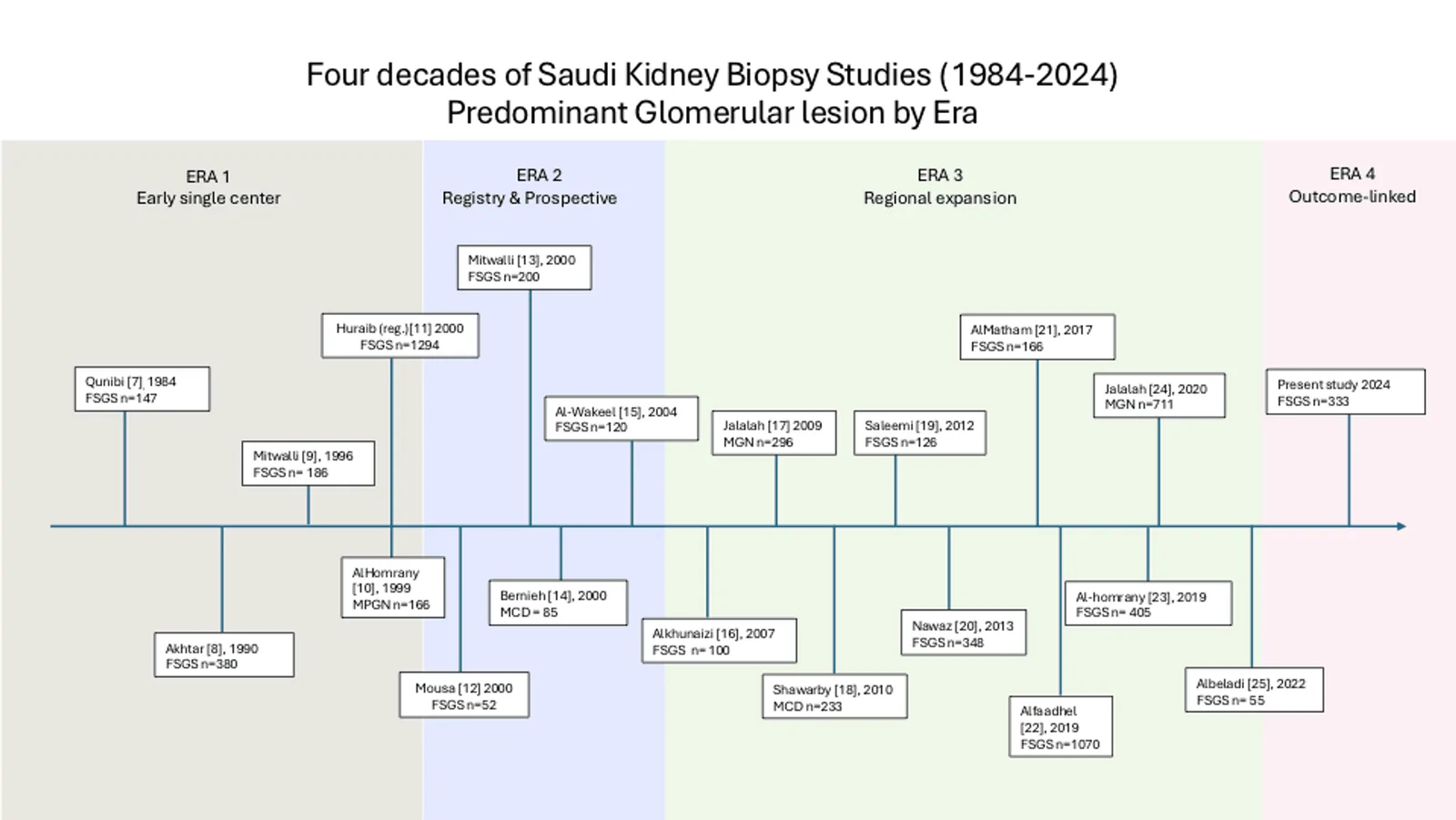
Timeline of published Saudi kidney biopsy Studies, 1984-2024, showing the predominant primary lesion in each series across four eras Each marker shows one published series (first author, reference number, and year), together with its predominant primary glomerular lesion and number of biopsies (n). Shaded bands indicate the four eras defined in Table 6; the present study is shown for comparison. Image credit: Hassan Homadi. Figure prepared using Microsoft PowerPoint (Microsoft Corporation, Redmond, WA, USA).

Our findings from a small single-center cohort are consistent with those of the largest international biopsy cohorts. In the VALIGA study, 1,147 European patients with IgA nephropathy were studied, and tubular atrophy and interstitial fibrosis independently predicted eGFR decline and reduced renal survival across the full spectrum of disease severity [32]. More directly comparable to our design, an analysis of 315 patients in the Nephrotic Syndrome Study Network (NEPTUNE) found that the degree of interstitial fibrosis predicted the composite outcome of a 40% eGFR decline or end-stage kidney disease, which closely matches our endpoint, independently of glomerular diagnosis, baseline eGFR, proteinuria, and medication use [33]. The association we found using routine pathology grading matches that observed with standardized scoring of whole-slide digital images in this North American consortium [33] and supports chronic tubulointerstitial damage as a prognostic marker.

These findings are relevant to the persistent lack of systematic, outcome-oriented data on glomerular disease in Saudi Arabia, where four decades of biopsy series have been concentrated in particular centers and regions, with differing methods and limited longitudinal follow-up. This limitation has been acknowledged by Saudi authors calling for a national renal biopsy registry [23]. This gap was highlighted again by the Kidney Disease in the National Guard (KIND) registry, whose rationale cites the lack of data on renal epidemiology and outcomes in the Kingdom [34]. That registry, however, identifies patients through ICD-10 coding across all forms of chronic kidney disease. This approach provides large numbers but lacks the detailed biopsy information that our data suggest is important for prognosis. The two approaches complement each other: administrative registries provide scale, while biopsy-based cohorts provide the pathological detail needed to link histology to outcomes. Together, they make the case for a dedicated GN registry combining structured histological scoring with prospective follow-up.

Strengths of this study include its 24-year observation period, complete light microscopy and immunofluorescence, with electron microscopy in selected cases, a distinct histopathological category of DKD+, and, to our knowledge, its status as one of the few local studies linking biopsy diagnosis to renal outcomes using multivariable analysis. However, several limitations should be acknowledged. First, the single-center retrospective design limits generalizability and introduces potential selection and referral bias, as biopsy practice and case mix vary between institutions. As a military tertiary center, our population may also differ from those of Ministry of Health and university hospitals in demographic and socioeconomic profiles and referral pathways. Furthermore, because patients with diabetes were generally biopsied only when atypical features were present, the observed frequencies of DKD+ and diabetic nephropathy reflect biopsy selection rather than true prevalence. Second, the outcome was based on a single assessment at one year. While this captures short-term functional decline, it does not describe the long-term course or progression, and our findings should not be extrapolated to long-term renal survival. Third, medication use reflected post-biopsy clinical prescribing rather than randomized exposure. Because treatment is directed to patients based on clinical presentation and risk, these variables are subject to confounding by indication, and their non-significant association cannot be considered evidence of an absence of benefit. Fourth, the regression was fitted in a complete-case cohort of 289 of 333 patients. Patients lost to follow-up (n = 19) may have had different renal outcomes, and 25 patients had incomplete covariate data; therefore, attrition bias cannot be excluded. Fifth, diagnoses were based on original pathology reports issued between 2000 and 2024 without central re-review, during which classification systems evolved. Finally, chronicity was graded visually rather than measured by digital image analysis, which limits reproducibility, although the scoring was based on routine diagnostic practice and supports clinical applicability.

## Conclusions

In this 24-year biopsy cohort, the severity of chronic histological damage independently predicted decline in kidney function at one year, alongside proteinuria and baseline eGFR. These findings show that chronicity on biopsy carries prognostic weight beyond the diagnostic level and that patients with mixed diabetic and non-diabetic pathology represent a high-risk group. While the range of diagnoses was consistent with the last four decades of Saudi biopsy literature, linking histological findings to outcomes adds something new and clinically meaningful and supports the need for GN-specific national data incorporating histological scoring and longitudinal outcomes.
